# Genome-wide characterization and expression analysis of the *ADF* gene family in response to salt and drought stress in alfalfa (*Medicago sativa*)

**DOI:** 10.3389/fpls.2024.1520267

**Published:** 2025-01-30

**Authors:** Mengmeng Shi, Yike Wang, Peng Lv, Yujie Gong, Qi Sha, Xinyan Zhao, Wen Zhou, Lingtao Meng, Zegang Han, Lingxiao Zhang, Yongwang Sun

**Affiliations:** ^1^ College of Agriculture and Biology, Liaocheng University, Liaocheng, China; ^2^ Shandong Binnong Technology Co., Ltd., Binzhou, China; ^3^ College of Agriculture and Biotechnology, Zhejiang University, Hangzhou, China; ^4^ State Key Laboratory of Crop Genetics and Germplasm Enhancement, College of Resources and Environmental Sciences, Nanjing Agricultural University, Nanjing, China

**Keywords:** actin-depolymerizing factor, gene family, alfalfa, abiotic stress, expression profile

## Abstract

The microfilament cytoskeleton, formed by the process of actin polymerization, serves not only to support the morphology of the cell, but also to regulate a number of cellular activities. Actin-depolymerizing factors (ADFs) represent a significant class of actin-binding proteins that regulate the dynamic alterations in the microfilament framework, thereby playing a pivotal role in plant growth and development. Additionally, they are instrumental in modulating stress responses in plants. The ADF gene family has been explored in various plants, but there was a paucity of knowledge regarding the *ADF* gene family in alfalfa (*Medicago sativa*), which is one of the most significant leguminous forage crops globally. In this study, a total of nine *ADF* genes (designated *MsADF1* through *MsADF9*) were identified in the alfalfa genome and mapped to five different chromosomes. A phylogenetic analysis indicated that the *MsADF* genes could be classified into four distinct groups, with members within the same group exhibiting comparable gene structures and conserved motifs. The analysis of the *K*a/*K*s ratios indicated that the *MsADF* genes underwent purity-based selection during its evolutionary expansion. The promoter region of these genes was found to contain multiple *cis*-acting elements related to hormone responses, defence, and stress, indicating that they may respond to a variety of developmental and environmental stimuli. Gene expression profiles analyzed by RT-qPCR experiments demonstrated that *MsADF* genes exhibited distinct expression patterns among different organs. Furthermore, the majority of *MsADF* genes were induced by salt and drought stress by more than two-fold, with *MsADF1*, *2/3*, *6*, and *9* being highly induced, suggesting their critical role in resistance to abiotic stress. These results provide comprehensive information on the *MsADF* gene family in alfalfa and lay a solid foundation for elucidating their biological function.

## Introduction

Microfilaments, formed by the polymerization of actin, constitute one of the three principal cytoskeleton components of eukaryotic cells. They are not only responsible for maintaining cell morphology through the three-dimensional structure, but also participate in regulating a number of cellular activities, including cell expansion, division, differentiation, organelle movement, and signal transduction (Porter and Day, 2016; Schaks et al., 2019). In cells, actin exists in two forms: monomeric globular actin (G-actin) and polymerized filamentous actin (F-actin). The ratio between the two populations varies with cell type and physiological status (Staiger, 2000). The latter, designated as microfilaments, represents the principal conduit through which actin exerts its biological functions (Pollard, 2016). The dynamics of actin within cells are controlled by dozens of actin-binding proteins (ABPs). In response to changes in growth stage or environmental conditions, ABPs regulate the activities of G-actin and F-actin, including polymerization, depolymerization, cross-linking and bundling. This ensures that the microfilament cytoskeleton undergoes highly dynamic changes, facilitating rapid cellular responses to intrinsic and extrinsic stimuli (Li et al., 2015; Porter and Day, 2016; Augustine et al., 2021).

Actin depolymerizing factor (ADF) is a kind of ABP with a small molecular weight (15~22 kDa) and a highly conserved sequence (Maciver and Hussey, 2002). The first ADF was isolated from chicken brain cells in 1980, and since then, ADFs have been identified in a diverse range of eukaryotic organisms, including fungi, animals, and plants (Bamburg et al., 1980; Gunning et al., 2015; Inada, 2017; Sun et al., 2023a). The biochemical activity of ADF is contingent upon its cellular concentration. Low concentrations of ADF promote severing or depolymerisation, whereas higher concentrations encourage G-actin nucleation and accelerate the release of Pi from ADP-Pi subunits in F-actin, along with the dissociation of branches formed by the actin-related protein 2/3 complex (Blanchoin et al., 2000; Andrianantoandro and Pollard, 2006). These biochemical activities make ADFs important participants in the regulation of dynamic changes in microfilaments within the cell, and thus they are involved in a number of cellular processes (Staiger, 2000; Pollard, 2016).

*ADF* genes are widely distributed in eukaryotic cells, with only one to three members in unicellular eukaryotes and animals, respectively. In contrast, higher plants have evolved a larger *ADF* gene family (Gunning et al., 2015; Sun et al., 2023a). To date, numerous plant *ADF* gene families have been identified, and the number of *ADF* genes in different species varies considerably. For example, common bean (*Phaseolus vulgaris*) has 9 (Ortega-Ortega et al., 2020), pigeon pea (*Cajanus cajan*) has 10 (Cao et al., 2021), *Arabidopsis thaliana*, rice (*Oryza sativa*), and tomato (*Solanum lycopersicum*) each has 11 (Feng et al., 2006; Ruzicka et al., 2007; Huang et al., 2012; Khatun et al., 2016), maize (*Zea mays*) has 13 (Huang et al., 2020), soybean (*Glycine ma*x) has 18 (Sun et al., 2023b), and wheat (*Triticum aestivum*) has 25 (Xu et al., 2021). Among plants, the expression characteristics and biological functions of the *Arabidopsis ADF* genes have been the subject of the most comprehensive study. Phylogenetic analysis demonstrates that the 11 *AtADF* genes can be classified into four groups (I~IV), with genes in each group displaying distinctive tissue expression patterns and differentiation in biochemical activities and biological functions (Feng et al., 2006; Ruzicka et al., 2007; Nan et al., 2017).

As sessile growth organisms, plants living in nature are subject to a range of biotic and abiotic stresses throughout their entire life cycle (Verma et al., 2016). Abiotic stress refers to the adverse effects on plants caused by non-living factors, including drought, water logging, salinity, extreme temperatures, and nutrient deficiency (Saijo and Loo, 2020). It has been demonstrated that plant *ADF* genes play a significant regulatory role in the response of plants to abiotic stress (Inada, 2017; Sun et al., 2023a). For example, *AtADF1* was found to plays an important role in the salt stress response pathway (Wang et al., 2021), while *AtADF5* has been confirmed to crucial for both of drought and cold tolerance ability of *Arabidopsis* (Qian et al., 2019; Zhang et al., 2021). In crop plants, *OsADF3* of rice, *ZmADF5* of maize, and *GmADF13* of soybean had been confirmed to plays a positive role in the drought response process (Huang et al., 2012; Liu et al., 2024; Wang et al., 2024), and *TaADF16* of wheat is crucial for the cold stress tolerance ability (Xu et al., 2021).

Alfalfa (*Medicago sativa*) is the most widely cultivated forage legume, with an area of cultivation exceeding 30 million hectares worldwide (Tang et al., 2021). It is a rich source of protein, vitamins, minerals, and numerous other nutrients, rendering it highly suitable for livestock feeding and earning it the designation of “king of forage” (Chen et al., 2021; Ma et al., 2022). Abiotic stresses, such as drought and salinity, have a significant detrimental impact on the growth and development of alfalfa, often resulting in considerable economic losses (Du et al., 2022; He et al., 2022). The exploration of stress-tolerance related genes in alfalfa is of great significance for the breeding of stress-tolerant cultivars, with the objective of ensuring their yield and quality. This study presents a systematic identification of the *ADF* gene family in alfalfa, accompanied by an investigation of their sequence characteristics, organs-specific expression, and expression patterns under salt and drought stress. The results of this study provide a foundation for the functional elucidation of *ADF* genes and stress-resistant breeding in alfalfa.

## Materials and methods

### Identification of the *ADF* genes in alfalfa

The complete genome, coding sequence, and protein sequence of alfalfa (cv. Zhongmu No.1) were obtained from the Figshare website (https://figshare.com/articles/dataset/Medicago_sativa_genome_and_annotation_files/12623960; Shen et al., 2020). The Hidden Markov Model profile of the ADF-H domain (PF00657) was obtained from the PFAM database (http://pfam.xfam.org/) and employed to identify ADF proteins via the HMMER software (http://hmmer.org/). The protein sequences of 11 AtADFs (Ruzicka et al., 2007) were obtained from the TAIR database (https://www.Arabidopsis.org/) and subjected to a BLASTP search against the alfalfa protein database, with an E-value threshold of < 10^-5^. The results of the HMMER and BLASTP searches were then merged, and any redundancies were removed manually. The non-redundant protein sequences were submitted to the NCBI CD-search (https://www.ncbi.nlm.nih.gov/Structure/cdd/wrpsb.cgi) and InterPro (https://www.ebi.ac.uk/interpro/) websites for further investigation to ascertain whether the ADF-H domain was present. Proteins that exhibited an intact ADF-H domain were designated as members of the ADF gene family and were subsequently named in accordance with their chromosomal location.

### Prediction of the physiochemical characteristics and subcellular location of MsADF proteins

The physicochemical characteristics of the MsADF proteins, including molecular weight (MW), isoelectric point (pI), instability index (InI), aliphatic index (AI), and grand average of hydropathy (GRAVY), were analyzed using the ProtParam tool which is available on the ExPASy website (https://web.expasy.org/protparam/). Subcellular localization predictions were conducted using the Wolf PSORT tool (https://wolfpsort.hgc.jp/).

### Chromosome location, gene duplication, and collinearity analysis

The genomic data of *Arabidopsis* and soybean were downloaded from Phytozome v13 database (https://phytozome-next.jgi.doe.gov/). The *MsADF* genes were mapped onto the alfalfa chromosomes based on their physical location. The duplication of these genes was analyzed and visualized using the TBtools software (Chen et al., 2020). A Multiple Collinear Scanning Toolkit (MCScanX) was employed to analyze the collinear blocks of *ADF* genes across alfalfa, *Arabidopsis*, and soybean, with the results visualized using TBtools. In order to assess the evolutionary divergence between duplicated *ADF* genes, the nonsynonymous substitution rate (*K*a) and the synonymous substitution rate (*K*s) of each gene pair were calculated using the *K*a/*K*s_Calculator 2.0 (Wang et al., 2010).

### Construction of the phylogenetic tree of the ADF proteins

Phylogenetic analysis was conducted on the ADF proteins from five plant species, including alfalfa (this study), *Arabidopsis* (Ruzicka et al., 2007), soybean (Sun et al., 2023b), rice (Feng et al., 2006), and maize (Huang et al., 2020). The analysis was performed using MEGA 11 software (https://www.megasoftware.net/). An unrooted phylogenetic tree was constructed using the Maximum Likelihood method with 1000 bootstrap replications, and pairwise deletions, based on the Poisson correction model (Kumar et al., 2016).

### Analysis of the genes structure, *cis*-acting elements, and conserved motif of *MsADF* genes

The GFF3 file of alfalfa was employed to analyze the exon-intron distribution of *MsADF* genes. The 2.0 kb sequence located upstream of the ATG start codon of each *MsADF* gene was extracted from the genome sequence and designated as their promoter. The *cis*-elements were analyzed using the PlantCARE website (http://bioinformatics.psb.ugent.be/webtools/plantcare/html/). The conserved motifs of the MsADF proteins were identified using the MEME website (http://meme-suite.org/) with the following parameters: a maximum of six motifs and optimal motif lengths of 6–100 amino acids. The TBtools software (Chen et al., 2020) was employed for the visualization of the distribution of exon-intron structures, *cis*-elements, and conserved motifs.

### Plant growth and stress treatments

The alfalfa (cv. Zhongmu No.1) seeds used in this study were stored at the College of Agriculture and Biology, Liaocheng University. The seeds were germinated on absorbent paper for a period of three days, after which the alfalfa seedlings were transferred to a plastic container containing a Hoagland nutrient solution for hydroponics. The seedlings were grown under a 16-h light/8-h dark photoperiod at a temperature of 24 ± 1°C and with 80% relative humidity (Du et al., 2022). The roots, nodules, stems, young leaves, mature leaves and flowers of mature alfalfa plants were collected separately and immediately frozen in liquid nitrogen and stored at –80°C until RNA extraction. To investigate the expression pattern of *MsADF* genes in response to salt and drought stress, seedlings (at the point when the third leaf was fully expanded) were exposed to nutrient solutions supplemented with 300 mM NaCl and 15% mannitol, respectively (Li et al., 2022). The sampling of the aboveground and underground parts of the plants was conducted at 0 h, 1 h, 3 h, 6 h, 12 h, and 24 h for each treatment, with the samples collected separately.

### RNA extraction and quantitative real-time PCR analysis

The total RNA was extracted using the Plant RNA Extraction Kit (TSINGKE, Cat. No. TSP401, China) in accordance with the instructions provided by the manufacturer. The RNA concentrations were found to range from 300 to 500 ng/µL, with 260/280 and 260/230 ratios approaching 2.0. The cDNA synthesis was conducted using the Prime Script RT Reagent Kit (TSINGKE, Cat. No. TSK301S, China), and the product was stored at –20°C until further use. The RT-qPCR was performed on a LightCycler^®^ 480 system (Roche, Basel, Switzerland) using SYBR Green qPCR kits (Vazyme, Cat. No. Q223, Nanjing, China). The relative gene expression was quantified using the 2^−ΔCt^ method, with MsActin (MsG0380016789) serving as the internal reference (Li et al., 2019). The data were analysed and visualized using GraphPad Prism 8.0 software (https://www.graphpad.com/). The results were based on the mean of three replicates, with statistical significance determined by Tukey’s pairwise comparison test. The gene-specific primers used in this study are listed in **Supplementary Table S1**.

## Results

### Genome-wide identification of *ADF* genes in alfalfa and characterization of their protein physicochemical properties

**Figure 1:**
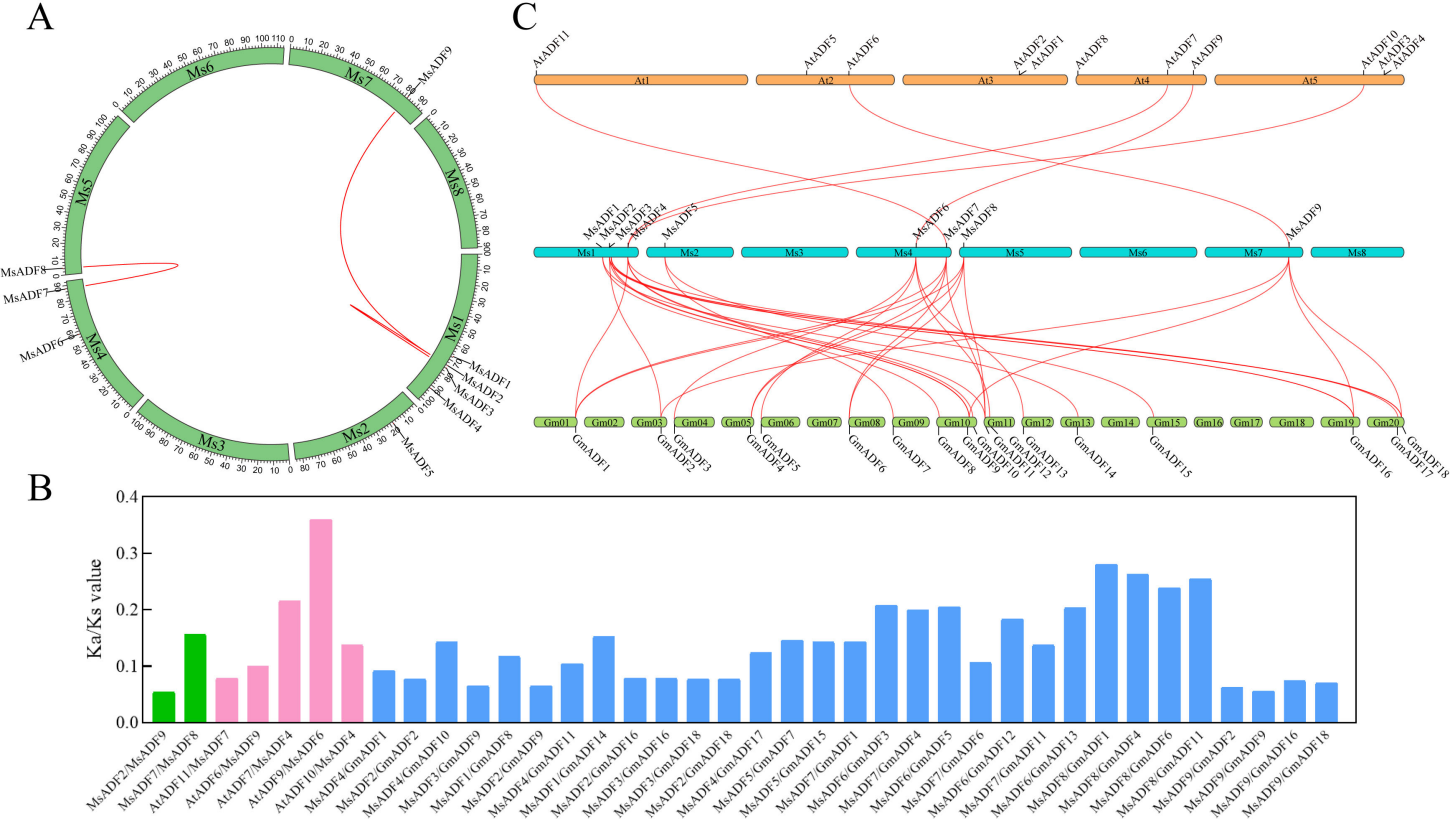
Chromosome distributions and the syntenic relationships of *MsADFs* in alfalfa. **(A)** The chromosomal location and interchromosomal relationship of alfalfa. The segmentally duplicated genes are connected by red lines. **(B)** The *K*a/*K*s values of *ADF* gene pairs for alfalfa-alfalfa, alfalfa-*Arabidopsi*s, alfalfa-soybean. **(C)** Syntenic maps of alfalfa, *Arabidopsis*, and soybean. The red lines highlight the syntenic *ADF* gene pairs.

Following the integration of HMMER and BLASTP search results, eleven non-redundant proteins were identified and subsequently analyzed for the presence of a conserved domain. Of these, nine proteins were confirmed to contain an intact ADF-H domain and were thus considered as members of the ADF gene family in alfalfa. These genes were designated MsADF1–MsADF9 in accordance with their chromosomal location (**Table 1**; **Figure 1**). The nucleotide sequence length of the MsADF genes exhibited considerable variation, with MsADF7 having a length of 895 bp and MsADF2 having a length of 3461 bp. The ADF proteins were observed to be generally shorter in length, with amino acid sequences ranging from 137 to 147 residues and molecular weights (MW) within the range of 15.77 to 16.90 kDa. The predicted pIs of the MsADF proteins ranged from 5.29 to 7.67, indicating that they tend to be neutral. The GRAVY values obtained were less than zero, indicating that these proteins possess hydrophilic characteristics. The InI values of all MsADF proteins were found to be greater than 40, indicating that they may be unstable. The AI values of 61.44 to 78.07 indicated that these proteins exhibited higher thermal stability (**Table 1**). Subcellular location prediction indicated that these proteins are predominantly localized to the chloroplast, followed by the cytoplasm, mitochondria, extracellular, and nuclear compartments, which suggests their functional roles in these organelles (**Supplementary Figure S1**). 

**Table 1:** Detailed information on the *MsADF* genes.

Name	Gene ID	Genomic length (bp)	Protein Length (aa)	MW (kDa)	pI	InI	AI	GRAVY
*MsADF1*	*MsG0180003726*	968	139	16.13	5.30	44.95	73.67	-0.506
*MsADF2*	*MsG0180004148*	3461	147	16.78	5.93	47.94	67.62	-0.485
*MsADF3*	*MsG0180004259*	3457	147	16.78	5.93	47.94	67.62	-0.485
*MsADF4*	*MsG0180005439*	1401	137	15.77	5.29	52.90	74.82	-0.301
*MsADF5*	*MsG0280007605*	1738	139	16.24	7.67	50.62	78.07	-0.420
*MsADF6*	*MsG0480021464*	969	143	16.45	6.58	47.32	69.58	-0.358
*MsADF7*	*MsG0480023628*	895	137	15.92	5.49	46.76	66.45	-0.473
*MsADF8*	*MsG0580024399*	1054	137	16.19	5.59	47.46	72.62	-0.463
*MsADF9*	*MsG0780040739*	3206	146	16.90	6.84	40.27	61.44	-0.664

### Chromosomal distribution and synteny of MsADF genes

The genomic distribution of the *MsADF* genes was determined by mapping the gene sequences onto their corresponding chromosomes. As illustrated in **Figure 1A**, the *MsADF* genes exhibited an uneven distribution across five of the eight alfalfa chromosomes, with one to four *MsADF*s present on each. Chromosome 1 has the highest number of *MsADF* genes (4), followed by chromosome 4 (2). Conversely, only one *MsADF* gene is located on chromosomes 2, 5, and 7. To examine gene duplication events within the *MsADF* family, a synteny analysis was conducted using TBtools software. A total of three pairs of genes resulting from segmental duplication were identified, with no evidence of tandem duplication (**Figure 1A**). To gain further insight into the evolutionary history of the ADF family genes in different plants, comparative syntenic maps were constructed for alfalfa, *Arabidopsis*, and soybean. The results demonstrated that 5 and 31 orthologous pairs were identified between alfalfa and *Arabidopsis*, and alfalfa and soybean, respectively (**Figure 1C**). Additionally, four *MsADF* genes (*MsADF4*, *6*, *7*, and *9*) exhibited a collinear relationship with those in *Arabidopsis* and soybean. It can be posited that these genes play an irreplaceable role in the evolution of the *ADF* gene family in higher plants. To gain insight into the evolutionary selection pressure exerted during the formation of the *ADF* gene family, the *K*a/*K*s values of *ADF* gene pairs were analyzed for alfalfa-alfalfa, alfalfa-*Arabidopsis*, and alfalfa-soybean (**Figure 1B**; **Supplementary Table S2**). The *K*a/*K*s values of these duplicated and orthologous gene pairs were all less than 1, indicating that *ADF* genes have been subjected to a potentially strong selective pressure during evolution.

### Phylogenetic relationships of the MsADF genes

**Figure 2:**
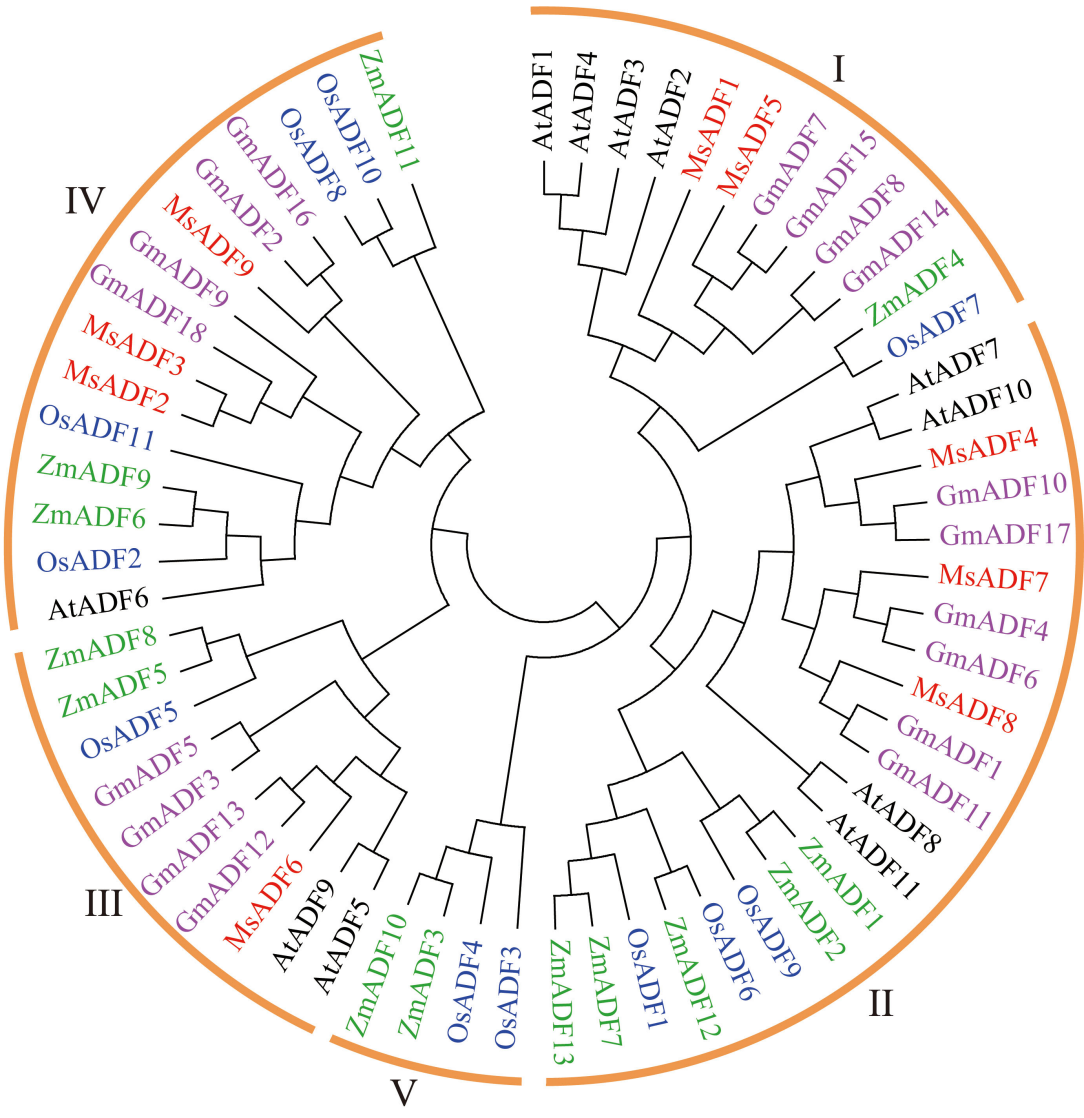
Phylogenetic analysis of ADF families across alfalfa, *Arabidopsis*, soybean, rice, and maize. Full-length protein sequences of ADFs were constructed using MEGA-11.0 based on the Maximum Likelihood method, bootstrap was 1,000 replicates.

To elucidate the evolution relationships among MsADF genes, a phylogenetic tree was constructed using the Maximum Likelihood method. The analysis incorporated 62 ADF proteins, comprising 9 from alfalfa, 11 from Arabidopsis, 11 from rice, 13 from maize, and 18 from soybean. As illustrated in **Figure 2**, the ADF proteins were classified into five distinct groups with varying levels of representation. Furthermore, group V, which consisted of only four monocot ADFs, was the only group that did not include ADFs from all five plant species. Group II comprised the largest number of ADF proteins (21), followed by Group IV (15), Group I (12) and Group III (10). Group II and IV each comprised three MsADFs, followed by groups I and III, which consisted of two and one member, respectively (**Table 2**).

**Table 2:** The total number of *ADF* genes in each group of *Arabidopsis*, alfalfa, soybean, rice, and maize.

Group	Arabidopsis	alfalfa	soybean	rice	maize
I	4	2	4	1	1
II	4	3	6	3	5
III	2	1	4	1	2
IV	1	3	4	4	3
V	0	0	0	2	2
Total	11	9	18	11	13

### Structural characteristics of the MsADF genes

**Figure 3:**
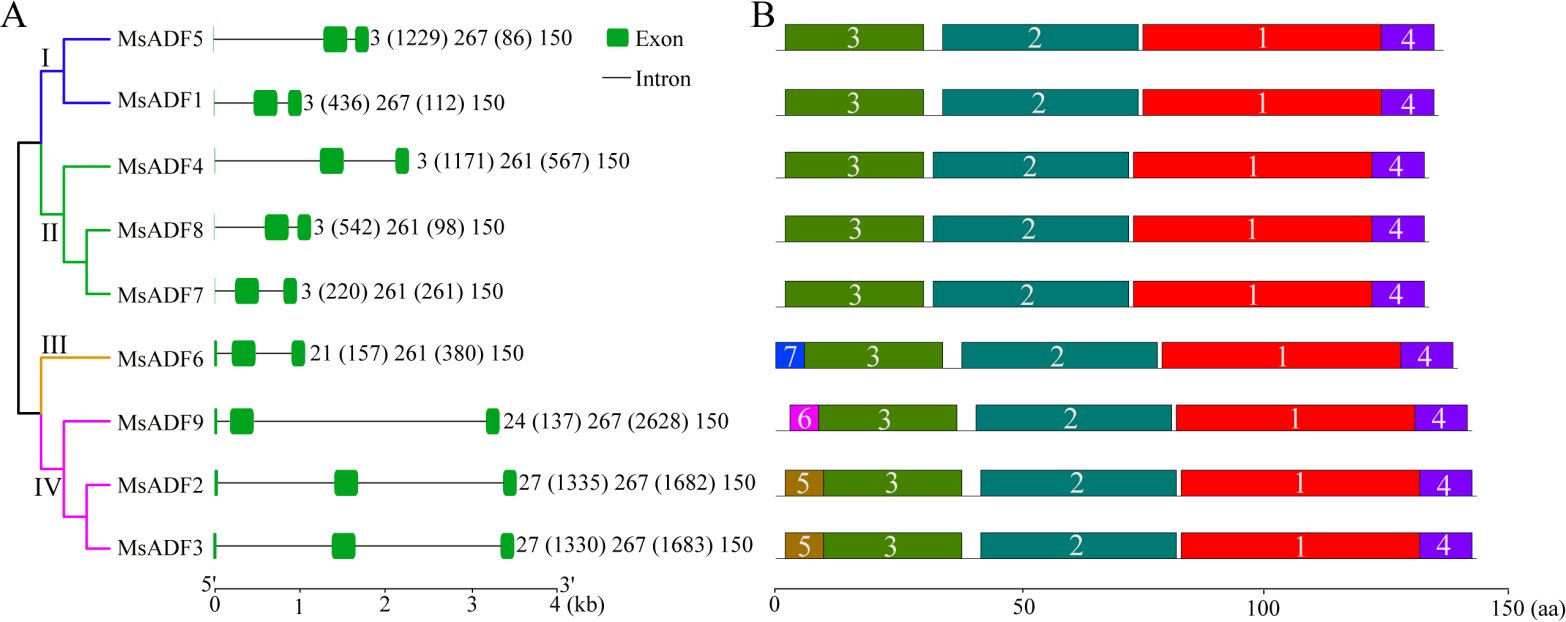
Gene structure and motif distribution of *MsADF* genes. **(A)** Exon-intron structures of *MsADF* genes. Green boxes and black lines indicating the exons and introns, respectively. **(B)** Distribution of conserved motifs of *MsADF* genes. The motifs were indicated in different colored boxes with different numbers and the sequence information for each motif was provided in **Supplementary Figure S3**.

Further analysis of the MsADF gene structures revealed that all genes shared a common architectural configuration, comprising two introns, a short exon at the 5’-terminus, a second exon of either 261-bp (in groups II and III) or 267-bp (in groups I and IV), and a 150-bp exon at the 3’-terminus (**Figure 3A**). The first exon of genes belonging to groups I and II was notably brief, consisting of only “ATG,” whereas those in groups III and IV exhibited a longer first exon, ranging from 21 to 27 bp. It is noteworthy that MsADF genes from Groups III and IV exhibited modifications in the conserved splicing sites (GT) following the ATG codon, resulting in splicing events occurring at subsequent splicing sites (GT). The observed variations in genomic length among the MsADF genes were primarily attributed to differences in intron length. Specifically, MsADF2, 3, 4, and 5 exhibited a longer first intron, exceeding 1 kb, while the second introns of the three genes from group IV (MsADF2, 3, and 9) displayed a markedly longer length, exceeding 1.5 kb, in comparison to the other six MsADF genes (**Figure 3A**).

### Characteristics of the protein sequence of MsADFs

The sequence identity among the MsADF proteins was found to be greater than 54.48% (between MsADF5 and MsADF6). Notably, the protein sequences of MsADF2 and MsADF3 were identical (**Supplementary Table S3**). A multiple protein sequence alignment revealed that all MsADFs contained both the ADF-H domain and regions associated with calmodulin- and actin-binding (**Supplementary Figure S2**). Moreover, the majority of MsADF proteins (with the exception of MsADF6 from Group III) exhibited a conserved serine residue, which may serve as a potential phosphorylation site. The MsADF proteins were found to possess seven conserved motifs, with Motif-1, Motif-2, Motif-3, and Motif-4 forming the core structure of the ADF-H domain. It was observed that members from Groups I and II only exhibited these four motifs (**Figure 3B**; **Supplementary Figure S3**). Motif-7 is specific to MsADF6, which is the sole member of Group III. In Group IV, Motif-5 and Motif-6 are present in the N-terminus of MsADF2/3 and MsADF9, respectively (**Figure 3B**; **Supplementary Figure S3**). The disparate motif distributions among MsADFs suggest that they may exhibit distinct biochemical activities.

In *Arabidopsis*, the two ADFs from group III (AtADF5 and AtADF9) have evolved to demonstrate the bundling function but not the classic depolymerizing activity. In contrast, the ADFs from group I demonstrated a stronger depolymerizing activity than those of groups II and IV (Nan et al., 2017). A number of amino acids were identified as being pivotal in the emergence of functional divergence among the AtADFs. The 11^th^ histidine (H11) was specific to group I AtADFs and was identified as a critical residue for the enhanced depolymerising activity. The sequence alignment revealed that two of the nine MsADFs, MsADF5 (belonging to Group I) and MsADF7 (belonging to Group II), possessed the H11 site (**Supplementary Figure S2**), indicating the potential for enhanced depolymerizing activity. Two lysines (K4 and K17) in the N-terminus of AtADF9 were shown to be crucial for its bundling function (Nan et al., 2017). It is noteworthy that these two homologous amino acids were also present in MsADF6, the sole member of group III (**Supplementary Figure S2**), indicating that a comparable biochemical activity differentiation may have occurred in this protein.

### Distribution of *cis*-acting elements in the promoter of *MsADF* genes

**Figure 4:**
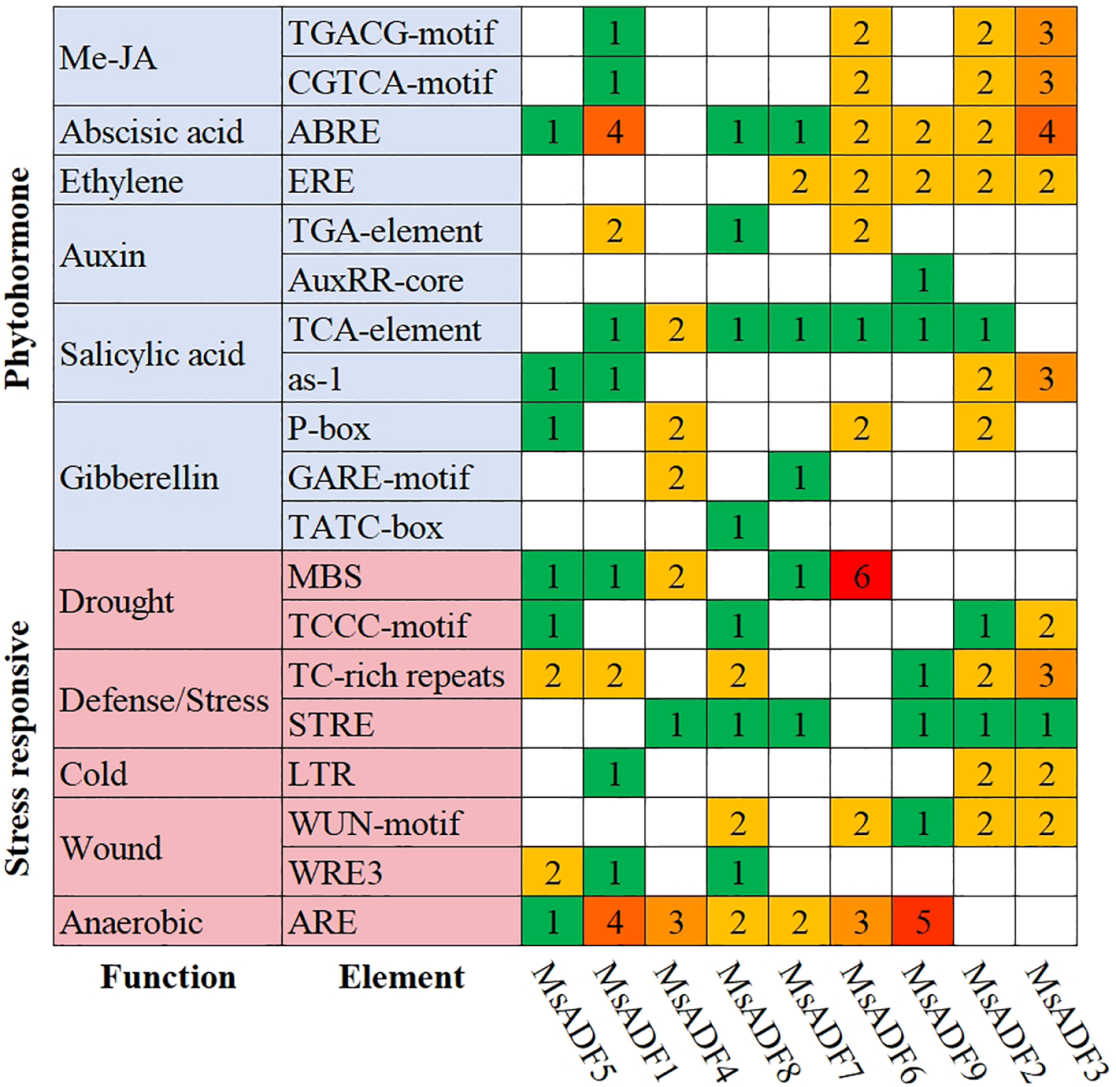
*Cis*-acting elements in the promoter regions of *MsADF* genes. The color and numerical value of the grid represent the quantities of various *cis*-acting elements present in the *MsADF* genes.

In order to investigate the potential regulatory elements controlling *MsADF* gene expression, 2.0 kb sequences upstream of the start codon were analyzed using the PlantCARE database. A total of 19 *cis*-acting elements related to hormone- and stress-responsiveness were identified (**Figure 4**). The number of abscisic acid-responsive elements was the highest among the hormone-responsive elements (17), followed by methyl jasmonate-responsive (16), salicylic acid-responsive (15), gibberellin-responsive (11) and auxin-responsive elements (6). *Cis*-acting elements involved in salicylic acid- and abscisic acid-responsiveness were identified in the promoter regions of nine and eight *MsADF* genes, respectively. All *MsADF* promoters were found to contain at least two type of hormone-responsive element. The *MsADF6* promoter, in particular, was observed to contain all the aforementioned hormone-responsive elements. The *MsADF* promoters were found to contain five distinct categories of stress-responsive elements. The most prevalent category of *cis*-acting elements was that of anaerobic-responsiveness, with 20 instances identified. This was followed by the defence/stress-responsive elements (18), drought-responsive elements (16), wound-responsive elements (13), and cold-responsive elements (5). These elements were identified in 7, 8, 8, 7, and 3 *MsADF* promoters, respectively. All *MsADF* promoters exhibited the presence of at least three distinct types of stress-responsive elements, with the *MsADF1* promoter demonstrating the presence of all five identified types of stress-responsive elements.

### Expression profiles of *MsADF* genes in different organs

**Figure 5:**
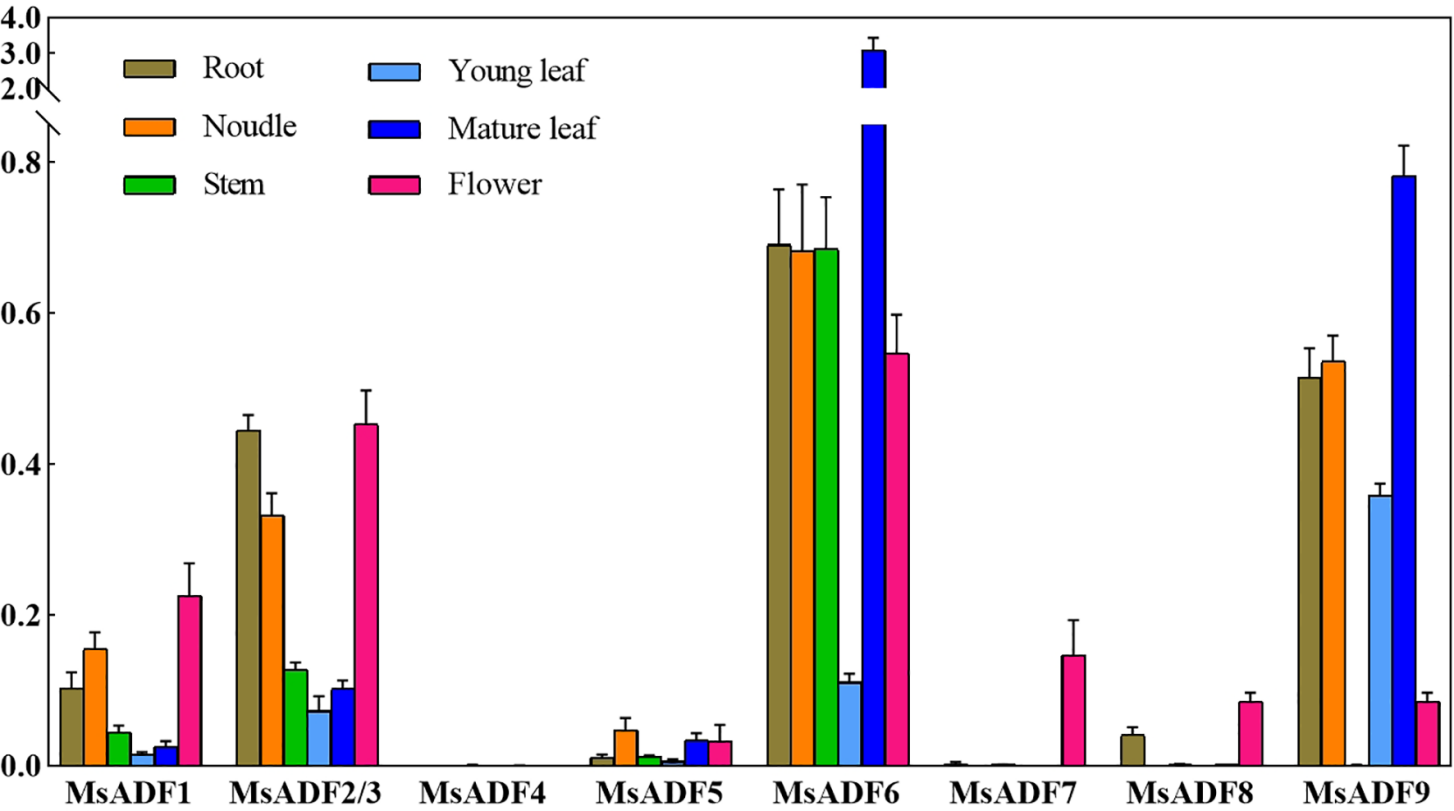
Expression Profiles of *MsADF* Genes. Expression levels of the nine MsADF genes via RT-qPCR in different organs: roots, nodules, stems, young leaves, mature leaves, and flowers. The different colors of the bars represent different organs. The standard error of the means of three independent replicates is represented by the error bars.

In order to gain insights into the spatial expression patterns of *MsADF* genes, their expression profiles in roots, nodules, stems, young leaves, mature leaves, and flowers were analysed by RT-qPCR (**Figure 5**). It should be noted that the coding regions, as well as the 5’ and 3’ untranslated regions of *MsADF2* and *MsADF3* are identical, thus rendering their expression levels indistinguishable. Consequently, the term “MsADF2/3” is used to represent the two genes’ expression levels. Of the nine genes under investigation, *MsADF4* was not expressed in any organ, while *MsADF6* and *MsADF9* exhibited strong expression in the majority of organs. The expression of *MsADF1*, *MsADF2/3*, *MsADF5*, and *MsADF6* was detected in all organs of alfalfa, while the expression levels of *MsADF1* and *MsADF5* were relatively low. In particular, *MsADF6* displays robust expression in all organs with the exception of young leaves, whereas *MsADF9* exhibits strong expression in all organs except stems and flowers. These observations underscore the pivotal role of these genes in the growth and developmental processes of alfalfa. It is noteworthy that all genes, with the exception of *MsADF4*, are expressed in flowers. Additionally, all genes, with the exception of *MsADF4* and *MsADF7*, are expressed in roots (**Figure 5**). The diverse expression patterns of *MsADF* genes suggest that they may play distinct roles in regulating organ development.

### Expression patterns of *MsADF* genes under salt and drought stress

**Figure 6:**
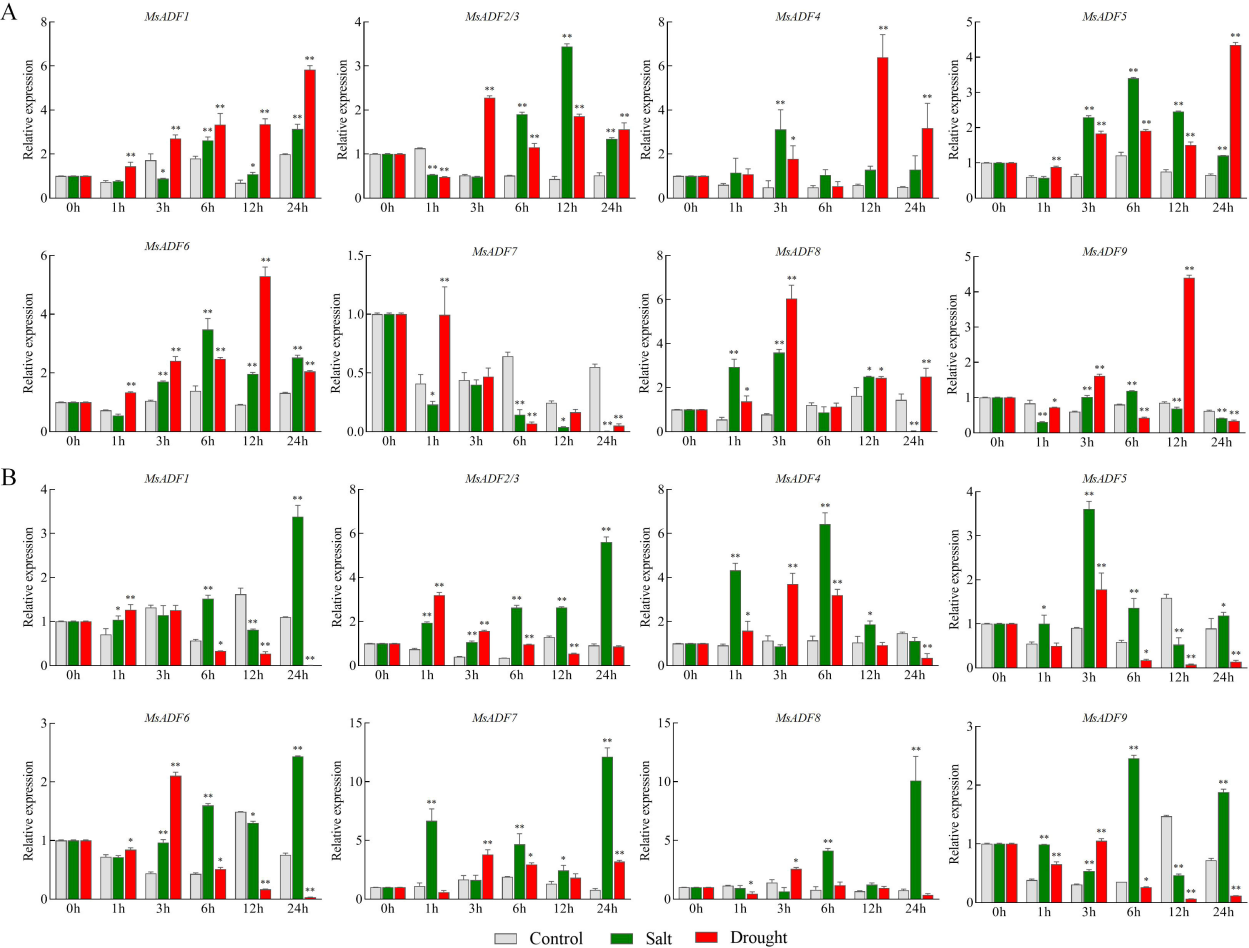
The relative expression levels of MsADF genes in underground and aboveground parts under salt and drought stresses. **(A)** Relative expression levels of MsADF genes in underground part under salt and drought stresses. **(B)** Relative expression levels of MsADF genes in aboveground part under salt and drought stresses. Actin gene was used as house-keeping gene in RT-qPCR. The results were calculated based on the mean of three replicates, and the statistical significance was determined through Tukey’s pairwise comparison test. ** indicates P < 0.01, and * indicates P < 0.05.

In order to investigate the potential function of *MsADF* genes in response to abiotic stress, the transcription levels of these genes in underground and aboveground parts were examined under salt and drought stress conditions using RT-qPCR (**Figure 6**). In the underground part, in response to salt stress, *MsADF7* exhibited rapid and sustained down-regulation, while the remaining genes displayed up-regulation at the initial or intermediate time point. The expression of *MsADF2/3*, *4*, and *8* was induced by salt stress, with a more than four-fold increase observed at 1 h, 3 h or 12 h post-treatment. Furthermore, the expression of *MsADF4*, *5*, *6*, *8*, and *9* exhibited a more than four-fold increase at certain time points, suggesting that they may play a role in the response to drought stress (**Figure 6A**). In the aboveground part, all genes were induced by salt stress, with the expression levels of *MsADF7* and *MsADF8* being particularly up-regulated by fourteen-fold and twelve-fold, respectively, at 24 hours post-treatment. Conversely, *MsADF4*, *6*, and *9* exhibited a peak at the 3-hour mark, subsequently declining. Under drought stress, the expression levels of the remaining genes in the aboveground part were down-regulated by more than one-fold at specific times, with most genes exhibiting a decrease in expression levels at 6 h, 12 h, and 24 h post-treatment. Conversely, *MsADF8* exhibited a decrease in expression level of more than one-fold at 1 h and 24 h post-treatment. In the aboveground part, the expression of most *MsADF* genes was inhibited after 6 hours of drought stress. Furthermore, the expression level of *MsADF1* demonstrated a persistent downward trend, reaching an extremely low and almost undetectable level at 24 hours (**Figure 6B**). It is noteworthy that, under salt stress, *MsADF1*, *7*, and *9* exhibited distinct expression patterns. These genes exhibited up-regulated expression in the aboveground part, while showing down-regulated or no significant change in the underground part. Conversely, under drought stress, the expression of *MsADF1*, *5*, and *8* was up-regulated in the underground part but down-regulated in the aboveground part (**Figure 6**), suggesting a specific functional localization of these genes.

## Discussion

The microfilament cytoskeleton is one of the most dynamic cellular components that respond constantly to environmental stimuli as they modulate organizations (Staiger, 2000). ADF is a major ABP that controls the rearrangement of microfilaments in eukaryotic cells, thereby participating in a vast number of cellular processes (Pollard, 2016). To date, the genome-wide identification of the *ADF* gene family has been accomplished in a multitude of higher plants, with the number of genes in different species ranging from a few to dozens (reviewed by Sun et al., 2023a). Nevertheless, a comprehensive analysis of the *ADF* gene family at the whole-genome level remains to be conducted in alfalfa, a crucial forage grass cultivated globally (Tang et al., 2021). In comparison to other gene families, such as MYB, bHLH, and WRKY, the *ADF* gene family in higher plants is relatively small in number (Wang and Li, 2017; Sun et al., 2023a; Wang et al., 2023; Gao and Dubos, 2024). In this study, a total of nine *MsADF* genes were identified in alfalfa, and an analysis of the protein sequence revealed that all of their proteins contained the conserved ADF-H domain and regions associated with calmodulin- and the actin-binding domains (**Supplementary Figure S2**). This number is approximately equivalent to the *ADF* family size in other plants, including *Arabidopsis* (11 members; Ruzicka et al., 2007), rice (11 members; Huang et al., 2012), tomato (11 members; Khatun et al., 2016), common bean (9 members; Ortega-Ortega et al., 2020), and pigeon pea (10 members; Cao et al., 2021). Gene duplication represents a fundamental source of new genes in the evolutionary process, contributing to the expansion of gene families and functional differentiation (Cannon et al., 2004). The primary duplication patterns for gene family expansion are tandem and segmental duplications, with the latter occurring with greater frequency due to the presence of duplicated chromosomal blocks (Panchy et al., 2016). This study revealed that three *MsADF* gene pairs underwent segmental duplication, with no evidence of tandem duplication. Furthermore, the *K*a/*K*s ratios of the three duplicated gene pairs were found to be less than 1 (**Figure 1B**; **Supplementary Table S2**), indicating the influence of purifying selection on the evolution of the *MsADF* gene family. Previous studies have documented the occurrence of segmental duplication events in the *ADF* families of wheat, *Arabidopsis*, maize, tomato, and soybean, with no instances of tandem duplication observed (Khatun et al., 2016; Nan et al., 2017; Huang et al., 2020; Xu et al., 2021; Sun et al., 2023b). It can therefore be postulated that the expansion of *ADF* gene families in higher plants is primarily attributed to segmental duplications. A collinearity analysis revealed that alfalfa shares five and 31 orthologous gene pairs with *Arabidopsis* and soybean, respectively (**Figure 1C**). The existence of orthologous gene pairs provides further evidence that the *ADF* gene family in plants exhibits a certain degree of conservation and may originate from common ancestral genes (Zheng et al., 2005). Notably, four *MsADF* genes (*MsADF4*, *6*, *7*, and *9*) demonstrated a collinear relationship with those in *Arabidopsis* and soybean (**Figure 1C**), indicating that they may have been conserved during evolution and perform analogous functions in disparate plant species.

Phylogenetic analysis is a valuable tool for predicting gene function, with genes belonging to the same clade often demonstrating similar biological functions (Kapli et al., 2020). In the present study, 62 ADF proteins in alfalfa, *Arabidopsis*, rice, maize, and soybean were classified into five groups (**Figure 2**), which is consistent with the classification of previous studies (Feng et al., 2006; Ruzicka et al., 2007; Huang et al., 2020; Sun et al., 2023b). With the exception of Group V, which exclusively comprised four monocot ADF proteins, the remaining four groups included ADF members from all five plant species. Furthermore, the number of ADFs in groups I–IV significantly differed between species, indicating that their ADF families underwent independent evolution and may have undergone functional differentiation, although ADFs in flowering plants are thought to have evolved from a common ancestor (Nan et al., 2017). Among higher plants, the function of *Arabidopsis ADF* genes has been the most thoroughly investigated, and dozens of *ADF* genes from other plants having been functionally characterized (see Sun et al., 2023a for a summary). The homologous relationship between *MsADF* and *ADF* genes from other plants may provide insights into their functional exploration. As a kind of evolutionary relic, the intron/exon arrangement carries the imprint of the evolution of a gene family, so analyzing exon-intron structures may provide insight into the evolutionary history of certain gene families (Wang et al., 2015). In this study, all *MsADF* genes were shown have three exons and two introns, and the length of three exons among different members were remarkably conserved (**Figure 3A**). This phenomenon had also been reported in *Arabidopsis*, rice, wheat, and soybean (Feng et al., 2006; Xu et al., 2021; Sun et al., 2023b). Moreover, the intron length of genes in the same clade were similar to each other, which further supports the analysis of the evolutionary relationship of *MsADF* genes.

In eukaryotic cells, the primary function of ADF has been identified as the acceleration of microfilament turnover through the facilitation of F-actin dynamics and the enhanced rate of G-actin departure from the F-actin end (Hussey et al., 2002). ADF has two potential binding sites for F-actin, one at the N-terminus and the other at the C-terminus (Tholl et al., 2011). Biochemical experiments demonstrated that several amino acid substitutions in the N-terminus resulted in significant variation of ADFs’ biochemical activity. For example, two members in group III (AtADF5 and AtADF9) have developed bundling rather than the typical depolymerizing activity; while ADFs belonging to group I (AtADF1, 2, 3, and 4) exhibit more pronounced depolymerizing activity compared to Groups II and IV (Nan et al., 2017). A motif analysis revealed that five MsADF proteins in groups I and II contain only the four motifs that constitute the ADF-H domain, whereas the other four members display a fifth motif at the N-terminus (**Figure 3B**; **Supplementary Figure S2**). This discrepancy in motif distribution suggests that MsADF may exhibit biochemical activity divergence. In AtADF9, two lysines (K4 and K17) at the N-terminus have been demonstrated to be essential for its bundling activity (Nan et al., 2017). It is noteworthy that these two homologous amino acids are also present in MsADF6, the only member of Group III. This suggests the possibility of a similar biochemical activity differentiation in this protein. In AtADFs, the H11 site is unique to Group I and plays a pivotal role in enhancing its depolymerizing activity (Nan et al., 2017). A further analysis of the sequence alignment reveals that two of the nine MsADFs, MsADF5 (belonging to Group I) and MsADF7 (belonging to Group II), contain the H11 site (**Supplementary Figure S2**), suggesting that they may have enhanced depolymerizing activity. The activity of ADF family proteins is also regulated by the N-terminal serine residue, a fact that has been verified in both vertebrates and maize (Agnew et al., 1995; Moriyama et al., 1996; Smertenko et al., 1998). Upon phosphorylation, ADF becomes inactivated and loses its F-actin-binding ability (Blanchoin et al., 2000). The phosphorylation of this serine residue is dependent on calcium-dependent calmodulin-like substances. Phosphorylation represents a pivotal step in ADF protein function and is closely associated with calcium signalling pathways. It enables the protein to respond to environmental and developmental cues, thereby remodelling the cytoskeleton (Allwood et al., 2001). With the exception of MsADF6, all other eight MsADF proteins contain a conserved serine residue in their N-terminus, which may be subject to phosphorylation, as has been reported in other ADFs. The N-terminal serine of MsADF6 was replaced with a threonine residue (**Supplementary Figure S2**), which had been demonstrated to undergo inhibitory phosphorylation by CDPK family members in plants and protists (Smertenko et al., 1998; Allwood et al., 2001). This substitution has been observed in numerous other ADFs belonging to group III, including AtADF5 and GmADF3, 5, 12, and 13 (Sun et al., 2023b), implying that the regulatory mechanism of group III ADFs’ biochemical activity may different from other groups. Biochemical analysis is needed in the future to determine the relationship between the phosphostatus of ADF and biological function.

The specific expression of *ADF* genes in different tissues and organs has been demonstrated in numerous plant species. The *ADF* genes belonging to the same group have been observed to exhibit a similar tissue-expression pattern (Ruzicka et al., 2007). In tomato, the primary expression of *SlADF1*, *SlADF3*, and *SlADF10* in flowers, particularly in stamens, suggests a potential involvement of these genes in flower and pollen development, as well as other regulatory factors (Khatun et al., 2016). In this study, apart from *MsADF4*, all other *MsADF* genes were expressed in flowers, with notably high expression levels of *MsADF2/3* from group IV and *MsADF6* from group III (**Figure 5**). This finding highlights the diversity and specificity of the *MsADF* gene family in the development of plant reproductive organs. Furthermore, *ADFs* are intimately linked with root formation and root hair development in plants (Bi et al., 2022). In *Arabidopsis*, *AtADF5* and *AtADF9* from group III, and *AtADF6* from group IV, display ubiquitous expression across various tissues. *AtADF5* displays robust expression in the root tip meristem, whereas *AtADF9* exhibits elevated expression in the root apical zone, trichomes, shoot apical meristem, and callus (Ruzicka et al., 2007). The present study demonstrates that *MsADF6*, which belongs to the same group as *AtADF5* and *AtADF9*, is strongly expressed in roots, nodules, old leaves, stems, and flowers (**Figure 5**). Similarly, *MsADF2/3* and *MsADF9*, which are grouped with *AtADF6*, exhibit widespread expression across different tissues. It is noteworthy that *MsADF9* demonstrates lower expression in stems compared to other tissues (**Figure 5**). These observations imply a significant role for these genes in the development of alfalfa, and the similarities in expression patterns between *MsADF* and *AtADF* genes suggest potential functional analogies.

ADFs play a pivotal role in plants’ response to external and internal stimuli by reorganizing the microfilament cytoskeleton, which alters cell morphology and serves as a key player in stress signaling pathways (Wang et al., 2011). Drought and salt are the most prevalent and severe abiotic stress for plant growth. In many species, the expression of *ADF* genes is induced by drought and salinity, and some *ADF* genes have been confirmed to having the potential to enhance plant resistance to stress (Sun et al., 2023a). In *Arabidopsis*, the expression of *AtADF1* was rapidly induced by salt stress, and transgenic *Arabidopsis* overexpressing *AtADF1* exhibited significantly increased survival rates under salt stress compared to the wild type (Wang et al., 2021). Drought stress can induce the expression of *AtADF5*, which in turn regulates the reorganization of microfilament structure through its F-actin bundling activity, thereby controlling stomatal movement and enhancing the plant’s ability to adapt to drought stress (Qian et al., 2019). The heterologous expression of *OsADF3* of rice, a gene exhibited significantly upregulation under various abiotic stress, in *Arabidopsis* has been demonstrated to enhance germination rate, primary root length, and seedling survival rate under conditions of drought stress (Huang et al., 2012). Overexpression of *ZmADF5* in maize resulted in a reduction in stomatal aperture, a decrease in water loss rate, an enhancement in ROS scavenging capacity, and an improvement in drought resistance in the plants (Liu et al., 2024). The enhancement of drought resistance by *GmADF13* is achieved through the modulation of osmoregulatory substance accumulation, the regulation of enzyme activities, and the regulation of stress tolerance-related gene expression (Wang et al., 2024). In this study, many *MsADF* genes were identified as being up-regulated under conditions of drought and salt stress. For instance, in the underground part, the expression levels of *MsADF4*, *5*, *6*, *8*, and *9* increased by more than four-fold in response to drought stress (**Figure 6A**); in the aboveground part, the expression levels of *MsADF7* and *8* increased by fourteen-fold and twelve-fold, respectively, under salt stress (**Figure 6B**). Concurrently, under salt and drought stress, the expression levels of *MsADF2/3*, *4*, and *6* were up-regulated in both the aboveground and underground parts (**Figure 6**). The identification of these genes offers significant references for subsequent functional research. However, in the underground part, the expression level of *MsADF7* was down-regulated under salt stress (**Figure 6A**). This phenomenon is analogous to the repression of *TaADF20* expression in wheat under cold stress (Xu et al., 2021). This phenomenon can be explained by the antagonistic relationships among *ADF* family genes. For instance, *AtADF9* has been shown to exert an antagonistic effect on *AtADF1* by modulating its degradation of actin. Conversely, when these proteins are ectopically expressed in tobacco cells, a contrasting effect is observed (Tholl et al., 2011).

The *cis*-acting elements within gene promoters are closely associated with gene expression patterns in response to different stresses (Cao et al., 2021). Huang et al. (2012) conducted a comprehensive analysis of the promoter structure and expression patterns of all 11 rice ADFs, revealing the presence of stress-related cis-acting elements within the 1-kb promoter regions. This finding suggests that certain *OsADF* genes are induced by diverse environmental stresses, including low temperature, drought, abscisic acid (ABA), and salt (Huang et al., 2012). Compared to other genes, the *MsADF7* promoter sequence exhibits the least number and types of stress-responsive *cis*-acting elements (**Figure 4**). This characteristic may uniquely determine the expression pattern and response mechanism of *MsADF7* to environmental stress. However, under specific time points of drought or salt stress, the expression level of *MsADF7* is found to be up-regulated in the aboveground part (**Figure 6B**), suggesting that *MsADF7* may play a role in the cross-talk of different signaling pathways under drought and salt stress (Yang et al., 2022). These findings underscore the complexity of the *MsADF* regulatory mechanism. Furthermore, the expression level of *MsADF1* exhibited a continuous downward trend, reaching an extremely low and almost undetectable level after 24 hours. The down-regulation of gene expression may serve as a signal for plants to perceive and respond to drought stress, thereby participating in signal transmission and response processes to help plants adapt to adverse environments. Additionally, this study identified that certain genes are involved in the expression of salt and drought response, demonstrating varied reactions. For instance, under salt stress, the expression of *MsADF1*, *7*, and *9* was up-regulated in the aboveground part, while their expression was down-regulated or did not differ significantly in the underground part (**Figure 6**), suggesting that their biological functions are location-specific. These findings suggest that the *MsADF* gene family may play a pivotal role in plants’ response to diverse abiotic factors and may serve as crucial regulatory factors for plants to cope with environmental stress. Further investigation into the functions and regulatory mechanisms of these genes is anticipated to provide invaluable theoretical and practical guidance for enhancing crop stress resistance.

## Conclusion

The present study identified a total of nine *MsADF* genes in the alfalfa genome. A phylogenetic analysis indicated that they could be classified into four groups, with members of the same group exhibiting analogous gene structure characteristics and conserved motifs. A number of *cis*-acting elements related to hormone- and stress- responsiveness were identified within their promoter region. Furthermore, gene expression profiles demonstrated that these *MsADF* genes exhibited disparate expression patterns in different organs. The RT-qPCR analysis demonstrated that the majority of the *MsADF* genes exhibited varying degrees of induction in response to salt and drought stress. Of particular note is the dramatic induction observed in *MsADF1*, *2/3*, *6*, and *9*. These genes represent promising candidates for further investigation into the molecular mechanisms underlying alfalfa stress resistance, as well as for the breeding of new alfalfa cultivars with enhanced stress tolerance. Such insights could potentially inform the genetic improvement of alfalfa resistance to abiotic stresses.

## Data Availability

The datasets presented in this study can be found in online repositories. The names of the repository/repositories and accession number(s) can be found in the article/**Supplementary Material**.
