# Supplementary material for: Genome-wide characterization and expression analysis of the ADF gene family in response to salt and drought stress in alfalfa (Medicago sativa)
Source: Front Plant Sci. 2025 Jan 30;15:1520267. doi: 10.3389/fpls.2024.1520267 (PMC11821967; doi:10.3389/fpls.2024.1520267)
Supplement: Supplementary file 1 [file DataSheet1.docx]

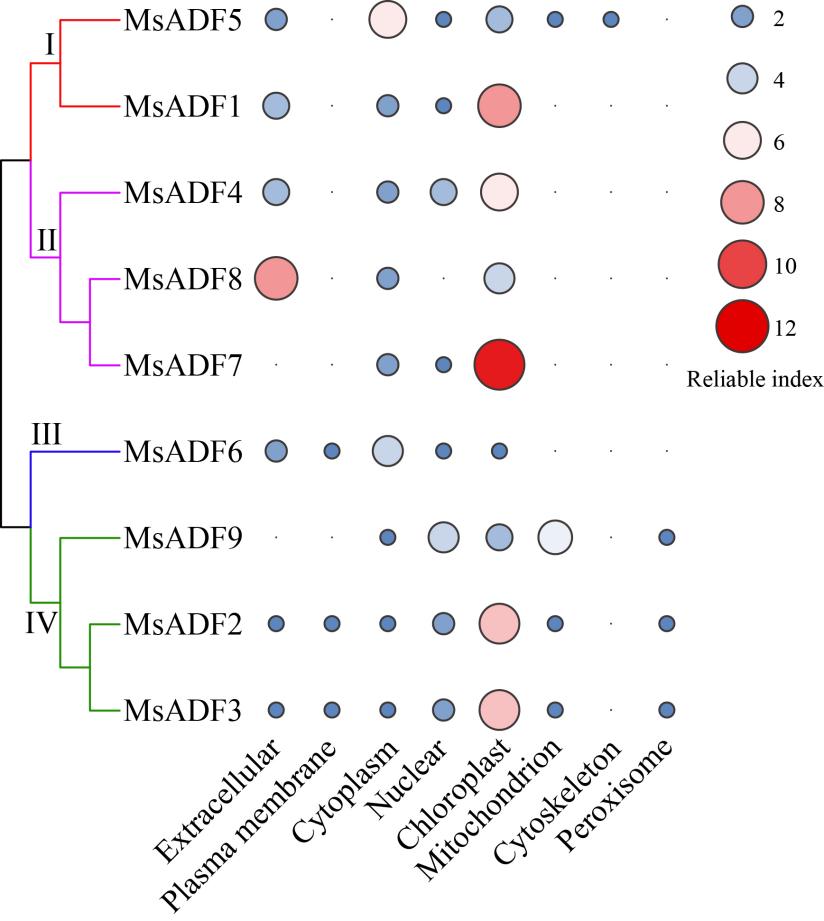


**Figure S1 The prediction of subcellular localization for MsADF proteins.** The color and the size of the circles indicate the reliability of the prediction results. The name of each protein is shown on the left. The site name for the predicted subcellular localization of each MsADF protein is shown at the bottom.


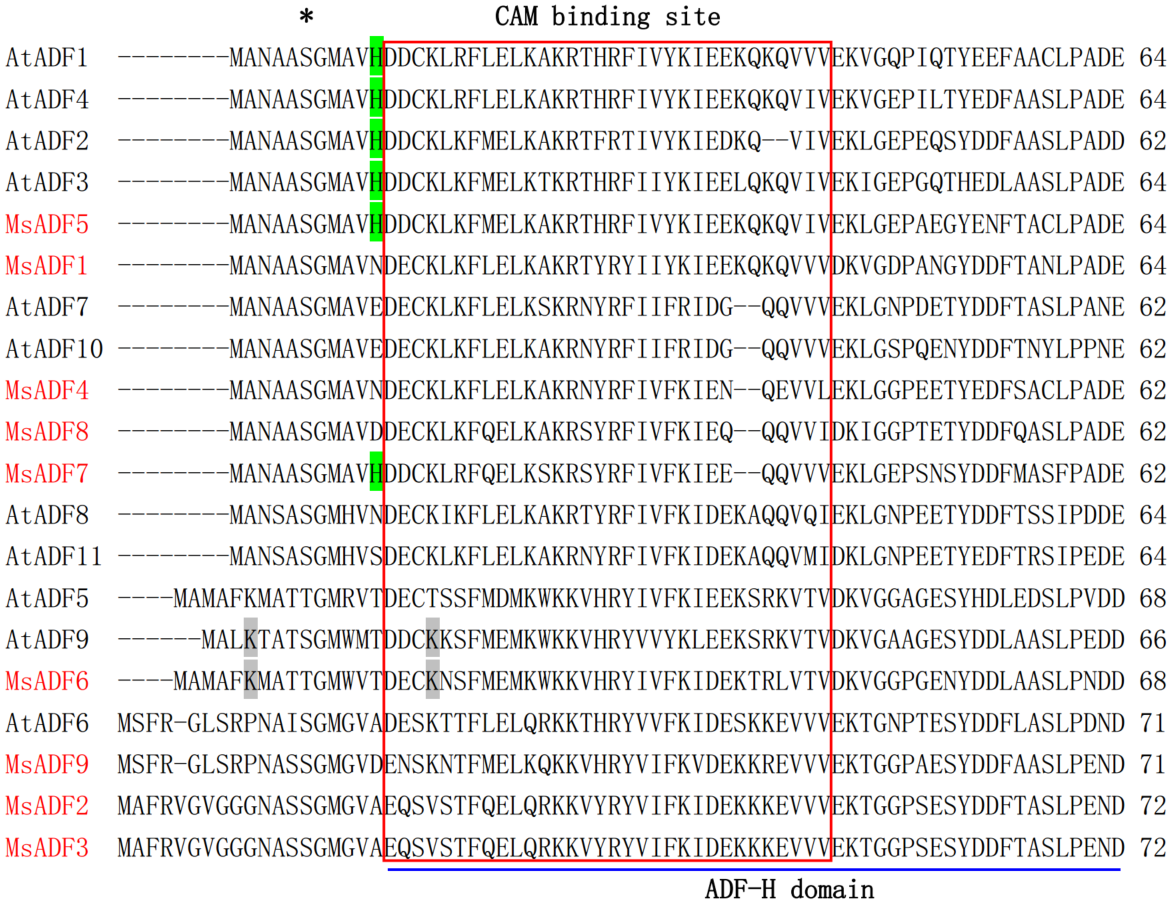


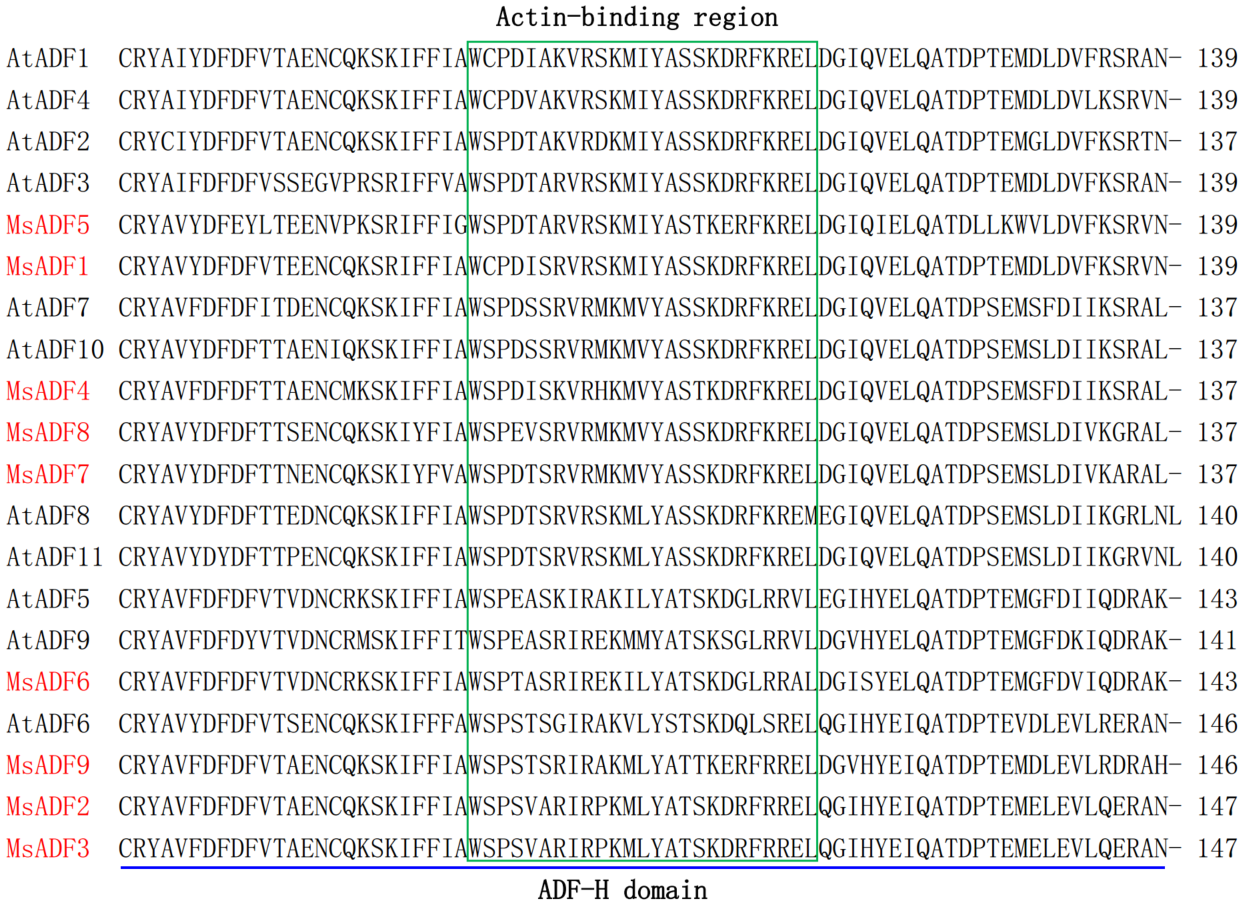


**Figure S2 The alignment of MsADF and AtADF protein sequences.** The asterisk indicates the putative Ser phosphorylation site. The red box indicates the putative CAM binding site and the green box indicates the amino acids essential for actin binding. The blue underline indicates the position of the ADF-H domain.


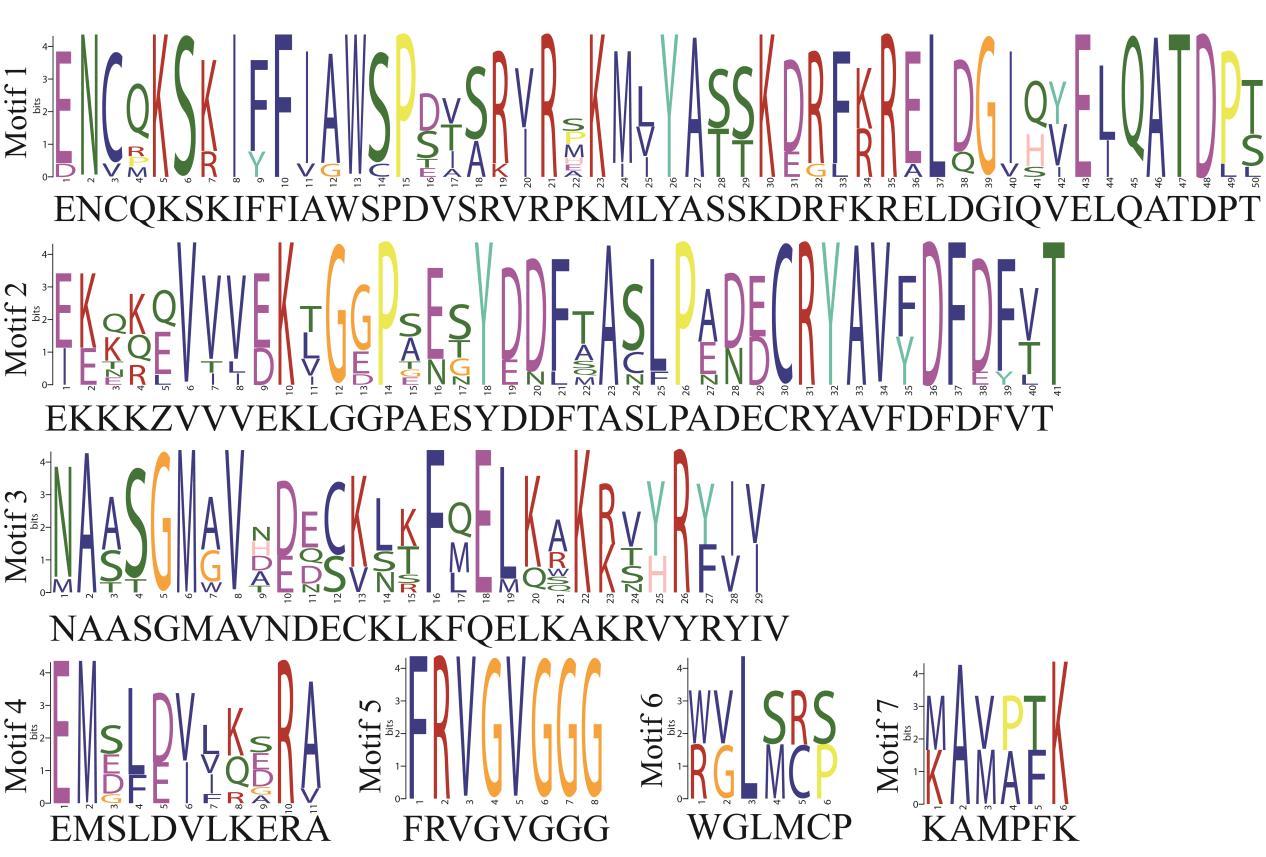


**Figure S3 The sequences of the seven conserved motifs identified from MsADFs.**

**Table S1 Primer sequences used for the RT-qPCR experiment of *MsADF* genes**

| Gene | Forward primer (5’-3’) | Reverse primer (5’-3’) | Product size |
| --- | --- | --- | --- |
| *MsActin* | AAGATGGCAGATGCTGAGGAT | CATGACACCAGTATGACGAGGTCG | 138 |
| *MsADF1* | TGACGAGTGTCGATATGCTGT | TTAATTGACACGGCTTTTGAACACAT | 235 |
| *MsADF2/3* | TCTTCTGGGATGGGTGTTGCT | GCAAGGATGCAGTGAAATCATCAT | 169 |
| *MsADF4* | CTACCTGCTGATGAGTGTCGT | TGCAACTCAACTTGAATGCCAT | 188 |
| *MsADF5* | GCAGTCCATGATGACTGCAAGT | ACTCATCAGCAGGAAGGCAT | 169 |
| *MsADF6* | ATGGCGATGGCTTTCAAAATGG | GCGGCAGTTGTCAACAGTGA | 255 |
| *MsADF7* | AGCCCTCCAACAGTTATGATGAT | TTAGAGGGCTCTCGCTTTCACAAT | 278 |
| *MsADF8* | GCTGATGAGTGTCGTTATGCTGT | CAATGTCCAAGCTCATCTCACTT | 237 |
| *MsADF9* | ATGTCTTTCAGAGGTCTCAGCC | GCGGCGAAATCGTCATAACTCT | 194 |

**Table S2 *K*a/*K*s values of the duplicated *ADF* gene pairs in alfalfa, *Arabidopsis*, and soybean**

| Duplicated gene pairs | *K*a | *K*s | *K*a/*K*s |
| --- | --- | --- | --- |
| *MsADF2/MsADF3* | 0 | 0 | no |
| *MsADF2/MsADF9* | 0.115877413 | 2.163902841 | 0.053550192 |
| *MsADF7/MsADF8* | 0.099258886 | 0.633283613 | 0.156736861 |
| *AtADF11/MsADF7* | 0.168139920 | 2.150973439 | 0.078169222 |
| *AtADF6/MsADF9* | 0.153871393 | 1.521153812 | 0.101154394 |
| *AtADF7/MsADF4* | 0.122329713 | 0.567787980 | 0.215449634 |
| *AtADF9/MsADF6* | 0.286822648 | 0.796496970 | 0.360105133 |
| *AtADF10/MsADF4* | 0.135433944 | 0.989975356 | 0.136805369 |
| *MsADF4/GmADF1* | 0.144114036 | 1.575573233 | 0.091467685 |
| *MsADF2/GmADF2* | 0.118887924 | 1.551244174 | 0.076640368 |
| *MsADF4/GmADF10* | 0.065799992 | 0.461571822 | 0.142556346 |
| *MsADF3/GmADF9* | 0.028242128 | 0.436273775 | 0.064734874 |
| *MsADF1/GmADF8* | 0.057049667 | 0.487008418 | 0.117143082 |
| *MsADF2/GmADF9* | 0.028242128 | 0.436273775 | 0.064734874 |
| *MsADF4/GmADF11* | 0.147763668 | 1.413548192 | 0.104533874 |
| *MsADF1/GmADF14* | 0.056869337 | 0.373744096 | 0.152161166 |
| *MsADF2/GmADF16* | 0.123646752 | 1.583945248 | 0.078062516 |
| *MsADF3/GmADF16* | 0.123646752 | 1.583945248 | 0.078062516 |
| *MsADF3/GmADF18* | 0.032800258 | 0.427223891 | 0.076775336 |
| *MsADF2/GmADF18* | 0.032800258 | 0.427223891 | 0.076775336 |
| *MsADF4/GmADF17* | 0.065799992 | 0.529414655 | 0.124288196 |
| *MsADF5/GmADF7* | 0.058785838 | 0.403390722 | 0.145729277 |
| *MsADF5/GmADF15* | 0.055133885 | 0.385129943 | 0.143156579 |
| *MsADF7/GmADF1* | 0.086227031 | 0.603107967 | 0.142971136 |
| *MsADF6/GmADF3* | 0.210927081 | 1.016024997 | 0.207600287 |
| *MsADF7/GmADF4* | 0.147214964 | 0.736651453 | 0.199843446 |
| *MsADF6/GmADF5* | 0.213032838 | 1.037871201 | 0.205259417 |
| *MsADF7/GmADF6* | 0.055075464 | 0.518629868 | 0.106194162 |
| *MsADF6/GmADF12* | 0.184201062 | 1.007256087 | 0.182874111 |
| *MsADF7/GmADF11* | 0.082741842 | 0.604915381 | 0.136782506 |
| *MsADF6/GmADF13* | 0.188259696 | 0.925912333 | 0.203323457 |
| *MsADF8/GmADF1* | 0.087856168 | 0.312836794 | 0.280837069 |
| *MsADF8/GmADF4* | 0.097407700 | 0.370088002 | 0.263201454 |
| *MsADF8/GmADF6* | 0.098259685 | 0.411509061 | 0.238778910 |
| *MsADF8/GmADF11* | 0.084355434 | 0.331428295 | 0.254520917 |
| *MsADF9/GmADF2* | 0.020808865 | 0.332019726 | 0.062673581 |
| *MsADF9/GmADF9* | 0.104256128 | 1.860473484 | 0.056037417 |
| *MsADF9/GmADF16* | 0.026895312 | 0.362847714 | 0.074122865 |
| *MsADF9/GmADF18* | 0.102011248 | 1.458148365 | 0.069959443 |

**Table S3 Protein sequence similarities among the MsADFs**

| Name | MsADF5 | MsADF1 | MsADF4 | MsADF8 | MsADF7 | MsADF6 | MsADF2 | MsADF3 | MsADF9 |
| --- | --- | --- | --- | --- | --- | --- | --- | --- | --- |
| MsADF5 | 100 |  |  |  |  |  |  |  |  |
| MsADF1 | 81.29 | 100 |  |  |  |  |  |  |  |
| MsADF4 | 71.53 | 75.18 | 100 |  |  |  |  |  |  |
| MsADF8 | 69.34 | 77.37 | 82.48 | 100 |  |  |  |  |  |
| MsADF7 | 71.53 | 75.18 | 78.83 | 86.13 | 100 |  |  |  |  |
| MsADF6 | 54.48 | 61.19 | 59.26 | 60.00 | 57.04 | 100 |  |  |  |
| MsADF2 | 55.07 | 62.32 | 54.74 | 58.39 | 57.66 | 61.64 | 100 |  |  |
| MsADF3 | 55.07 | 62.32 | 54.74 | 58.39 | 57.66 | 61.64 | 100 | 100 |  |
| MsADF9 | 58.39 | 61.31 | 59.56 | 59.56 | 59.56 | 68.15 | 80.27 | 80.27 | 100 |
